# Association between *hTERT* rs2736100 polymorphism and sensitivity to anti-cancer agents

**DOI:** 10.3389/fgene.2013.00162

**Published:** 2013-08-26

**Authors:** Julie Kim, Yava L. Jones-Hall, Rongrong Wei, Jamie Myers, Yuan Qi, Gregory T. Knipp, Wanqing Liu

**Affiliations:** ^1^Department of Medicinal Chemistry and Molecular Pharmacology, College of Pharmacy, Purdue UniversityWest Lafayette, IN, USA; ^2^Department of Comparative Pathobiology, College of Veterinary Medicine, Purdue UniversityWest Lafayette, IN, USA; ^3^Department of Medicine, The University of ChicagoChicago, IL, USA; ^4^Department of Computer Science, Purdue UniversityWest Lafayette, IN, USA; ^5^Department of Industrial and Physical Pharmacy, College of Pharmacy, Purdue UniversityWest Lafayette, IN, USA; ^6^Purdue University Center for Cancer ResearchWest Lafayette, IN, USA

**Keywords:** TERT, polymorphism, rs2736100, anticancer drug, sensitivity

## Abstract

**Background:** The rs2736100 single nucleotide polymorphism (SNP) is located in the intron 2 of human telomerase reverse transcriptase (*hTERT*) gene. Recent genome-wide association studies (GWAS) have consistently supported the strong association between this SNP and risk for multiple cancers. Given the important role of the *hTERT* gene and this SNP in cancer biology, we hypothesize that rs2736100 may also confer susceptibility to anti-cancer drug sensitivity. In this study we aim to investigate the correlation between the rs2736100 genotype and the responsiveness to anti-cancer agents in the NCI-60 cancer cell panel.

**Methods and Materials:** The *hTERT* rs2736100 was genotyped in the NCI-60 cancer cell lines. The relative telomere length (RTL) of each cell line was quantified using real-time PCR. The genotype was then correlated with publically available drug sensitivity data of two agents with telomerase-inhibition activity: Geldanamycin (HSP90 inhibitor) and RHPS4/BRACO19 (G-quadruplex stabilizer) as well as additional 110 commonly used agents with established mechanism of action. The association between rs2736100 and mutation status of *TP53* gene was also tested.

**Results:** The C allele of the SNP was significantly correlated with increased sensitivity to RHPS4/BRACO19 with an additive effect (*r* = −0.35, *p* = 0.009) but not with Geldanamycin. The same allele was also significantly associated with sensitivity to antimitotic agents compared to other agents (*p* = 0.003). The highest correlation was observed between the SNP and paclitaxel (*r* = −0.36, *p* = 0.005). The telomere length was neither associated with rs2736100 nor with sensitivity to anti-cancer agents. The C allele of rs2736100 was significantly associated with increased mutation rate in *TP53* gene (*p* = 0.004).

**Conclusion:** Our data suggested that the cancer risk allele of *hTERT* rs2736100 polymorphism may also affect the cancer cell response to both TERT inhibitor and anti-mitotic agents, which might be attributed to the elevated telomerase-independent activity of *hTERT*, as well as the increased risk for *TP53* gene mutagenesis conferred by the polymorphism. Detailed mechanisms need to be further investigated.

## Introduction

Telomerase is a ribonucleoprotein enzyme complex that maintains telomere ends by addition of telomeric repeats to the 3′ hydroxyl ends of DNA strands in the telomeric region (Artandi and DePinho, 2010). Telomeres are essential for protecting chromosomal ends from degradation and preventing inappropriate fusion and rearrangements. Human telomerase is composed of two important subunits: telomerase RNA (hTERC or hTR) which serves as the template for telomere elongation and telomerase reverse transcriptase (hTERT) which possesses the catalytic activity to synthesize DNA from the RNA template (Newbold, 2002; Shay and Wright, 2005; Artandi and DePinho, 2010). Telomerase activity has been observed in about 90% of human cancers and is strongly associated with cellular immortalization, which is thought to be a prerequisite for malignant transformation (Newbold, 2002; Artandi and DePinho, 2010).

Telomerase role in maintenance of telomeres is strongly associated with cellular immortalization, and emerging evidence reports that telomerase may have the capacity to directly regulate cancer-promoting pathways through several mechanisms, including telomere elongation-independent activity (Stewart et al., 2002; Blasco and Hahn, 2003; Smith et al., 2003; Cong and Shay, 2008; Parkinson et al., 2008; Mukherjee et al., 2011). For this reason, telomerase is considered an ideal target for anticancer therapeutics and several strategies for telomerase inhibition are in development and have entered clinical trials (Ruden and Puri, 2013). One approach involves directly inhibiting hTERT catalytic activity using small molecule inhibitors, immunotherapy, and gene therapy (Ruden and Puri, 2013). Several approaches to targeting telomerase activity have been developed including: G-quadruplex stabilizers, tankyrase inhibitors, and HSP90 inhibitors. A G-quadruplex is a higher order structure that protects telomeric 3′-overhangs from being accessed by telomerase. G-quadruplex ligands inhibit telomerase activity by stabilizing G-quadruplex and may also trigger telomere uncapping by causing dissociation of telomere-associated proteins. Tankyrase and HSP90 inhibitors block telomerase activity by inhibiting binding of telomerase-associated proteins, leading to telomere uncapping and cell apoptosis (Ruden and Puri, 2013).

Given the important role of hTERT in cancer biology, many researchers sought to investigate whether genetic variation at the *hTERT* gene is associated with cancer development (Fujiwara et al., 2000; Shete et al., 2009; Din et al., 2011; Hu et al., 2011; Kinnersley et al., 2012; Mocellin et al., 2012; Zou et al., 2012). The rs2736100 single nucleotide polymorphism (SNP) is one of the most consistently identified variants of the *hTERT* gene associated with cancer risk in multiple studies (Shete et al., 2009; Hu et al., 2011; Kinnersley et al., 2012; Mocellin et al., 2012; Zou et al., 2012). Thirty-two studies thus far have reported a statistically significant association between C allele of rs2736100 and susceptibility to bladder, lung, pancreatic and CNS tumors, but interestingly associated with a decreased risk for testicular cancer (Mocellin et al., 2012). These studies strongly suggested that TERT genetic variation confers risk to cancer pathogenesis. Whether this variation affects cellular response to anticancer agents and subsequently leads to inter-individual variability in clinical outcomes remains uninvestigated thus far. We hypothesize in this study that rs2736100 may confer susceptibility to the sensitivity to anticancer treatment especially agents with telomerase inhibition activities. To corroborate this hypothesis, we chose the NCI-60 cancer cell panel as an *in vitro* model to evaluate the relationship between rs2736100 and 2 agents with telomerase inhibition activity and additional 110 commonly used anticancer agents with known mechanism of action.

## Materials and methods

### DNA extraction and genotyping

DNA samples of the NCI-60 cell panel were provided by the Pharmacogenetics of Anti-cancer Agents Research Group (PAAR). DNA was isolated from NCI-60 cancer cells (*n* = 58) in our previous studies (Liu et al., 2007, 2009). The rs2736100 SNP was genotyped using TaqMan® SNP genotyping assays (Applied Biosystems, Foster City, CA). Amplification for rs2736100 was conducted on a BioRad CFX96 Real-Time PCR Detection System (Bio-Rad, Hercules, CA) with the following program: 95°C for 10 min, followed by 40 cycles of 92°C for 15 s and 60°C for 1 min. Data were analyzed with BioRad CFX Manager software. A total of 9 samples with an ambiguous genotyping call in the Taqman assay were also PCR amplified and sequenced to confirm the genotypes.

### Measurement of relative telomere length (RTL)

The copy number of the telomere repeat (T) relative to the mean copy number (S) of two single copy control genes *36B4* (encoding acidic ribosomal phosphoprotein P0) and *HBB* (beta-globin) were determined by real-time PCR. The result was expressed as the T/S ratio. PCR primer sequences were according to the previous report by Capezzone et al. (2008). All samples were run in triplicates in a ViiA-7 Real-Time PCR system (Applied Biosystems, CA, USA). In each run, a standard curve and a negative control were included. The final concentration of reagents in the PCR were 1× SYBR Green Supermix, 300 nM Telomere and *36B4* primers or 400 nM *HBB* primers and 2 ng DNA for a final volume of 7.5 uL. Thermal cycling profile for the telomere amplification was: 95°C for 10 min, followed by 40 cycles of 95°C for 15 s and 54°C for 1 min. For *36B4* and *HBB*, amplification was 95°C for 10 min, followed by 40 cycles of 95°C for 15 s and 60°C for 1 min. To ensure the amplification specificity, a melting curve program was set as follows: 95°C for 15 s, 60°C for 1 min, and 95°C for 15 s, right after the PCR cycles.

### Extraction of cytotoxicity data and *TP53* gene mutation data from the NCI-60 database

Publically available GI50 (molar concentrations expressed as log_10_[IC50]) data of two agents with telomerase inhibition activity, a HSP90 inhibitor Geldanamycin (NSC330507) and G-quadruplex stabilizer RHPS4/BRACO19 (NSC714187), as well as 111 drugs with known mechanism of actions (Scherf et al., 2000) were obtained from the NCI-60 database as described in our previous study (Liu et al., 2009). The 111 drugs were clustered into 6 main groups based on their cytotoxic activity: alkylating agents (*n* = 35), antimitotic agents (*n* = 16), DNA antimetabolites (*n* = 15), RNA/DNA antimetabolites (*n* = 19), topoisomerase I inhibitors (*n* = 12) and topoisomerase II inhibitors (*n* = 14). Due to the low variability in the GI50 data, an antimitotic drug NSC49842 was removed from the list. Therefore, a total of 110 common drugs were included in the analysis.

The method for drug sensitivity screening in the cancer cell line panel was described in the NCI60 database (http://dtp.nci.nih.gov/branches/btb/ivclsp.html). Note that the cytotoxicity assays were not performed in our study. The protocol was included in case one would follow to replicate the study. Briefly, depending on the doubling time of each individual cell line 5000–40,000 cells/well were grown in 96-well microtiter plates in 100 μL at RPMI 1640 medium containing 5% fetal bovine serum and 2 mM L-glutamine. Cells were then incubated at 37°C, 5% CO_2_, 95% air, and 100% relative humidity for 24 h prior to drug treatment. After incubation, two plates of each cell line were fixed *in situ* with trichloroacetic acid (TCA). This was used to be a measurement of the cell population for each cell line at the time of drug addition (Tz). Each experimental drug was first solubilized in dimethyl sulfoxide (DMSO) at the concentration that is 400-fold the desired final maximum test concentration. Solution was then aliquoted and stored frozen until use. For each drug screening, one aliquot of frozen concentrate was thawed and diluted to twice the desired final maximum test concentration with complete medium containing 50 μg/ml gentamicin. A total of five drug concentrations and control were made by 10-fold or ½ log serial dilutions. For each concentration, 100 μl of the drug dilution were aliquoted into the appropriate microtiter wells that already contained 100 μl of medium, which resulted in the required final drug concentrations. Medium in each plate was then mixed and incubated at 37°C, 5% CO_2_, 95% air, and 100% relative humidity for 48 h. For the detection of cytotoxicity, the assay was terminated by the addition of cold TCA for adherent cells. Cells were then fixed *in situ* by the gently adding 50 μl of cold 50% (w/v) TCA (final concentration, 10%) and incubated for 60 min at 4°C. After incubation, the supernatant was discarded, and the plates were washed five times with tap water and air dried. Cells were then stained by incubating in the plate for 10 min at room temperature by adding sulforhodamine B (SRB) solution (100 μl) at 0.4% (w/v) in 1% acetic acid in each well. After staining, unbound dye was discarded, followed by washing five times with 1% acetic acid and air dried. Bound stain was subsequently solubilized by incubating with 10 mM trizma base, and the absorbance was read on an automated plate reader at a wavelength of 515 nm. For suspension cells, the assay was terminated by fixing settled cells at the bottom of the wells by gently adding 50 μl of 80% TCA (final concentration, 16% TCA). The rest procedure was the same. The percentage growth was calculated at each of the drug concentrations, by using the seven absorbance measurements [time zero (Tz), control growth (C), and test growth in the presence of drug at the five concentration levels (Ti)], Percentage growth inhibition was calculated as:

$$\begin{array}{l} “[\left(\text{Ti}-\text{Tz}\right)/\left(\text{C}-\text{Tz}\right)]\;\times \;100\text{ for concentrations for which Ti}\geq \text{Tz}\\ \left[\left(\text{Ti}-\text{Tz}\right)/\text{Tz}\right]\;\times \;100\text{ for concentrations for which Ti}<\text{Tz}. \end{array}$$

Three dose response parameters (GI50, TGI, and LC50) were calculated for each experimental agent. Growth inhibition of 50% (GI50) was calculated from [(Ti − Tz)/(C − Tz)] × 100 = 50, which was the drug concentration resulting in a 50% reduction in the net protein increase (as measured by SRB staining) following treatment as compared to the control cells. The drug concentration resulting in total growth inhibition (TGI) was calculated from Ti = Tz. The LC50 (concentration of drug resulting in a 50% reduction in the measured protein at the end of the drug treatment as compared to that at the beginning) indicating a net loss of cells following treatment was calculated from [(Ti − Tz)/Tz] × 100 = −50. Values were calculated for each of these three parameters if the level of activity was reached; however, if the effect was not reached or was exceeded, the value for that parameter was expressed as greater or less than the maximum or minimum concentration tested.” (http://dtp.nci.nih.gov/branches/btb/ivclsp.html).

Mutation status of *TP53* gene was downloaded from the NCI-60 database using the accession ID MT2924. The *TP53* mutations were identified using PCR-sequencing in previous study (Ikediobi et al., 2006).

### Statistics

Comparison of the genotype frequency between our data and the Hapmap data as well as the test for Hardy–Weinberg Equilibrium (HWE) were conducted using Chi-square test. Associations between SNP genotype and cytotoxicity data as well as relative telomere length (RTL) were first screened using One-Way ANOVA with a statistical significance set to *p* = 0.05. A *post-hoc* linear regression was performed to identify the best genetic model. In doing so, SNP genotype was correlated with RTL or drug cytotoxicity data by assigning each genotype to 0, 1, and 2 (additive model), 0, 0, and 1 (receive model) or 0, 1, and 1 (dominant model) according to the number of C allele (Codd et al., 2013). Association between SNP genotype and the cytotoxicity data of a specific group of drugs was tested using Fisher's exact test based on a 2 × 2 contingency table, by comparing the number of positive correlation (*p* < 0.05) in a given group compared with that of the remaining drugs. Correlation between RTL cytotoxicity data was performed using linear regression. RTL data were log transformed (+log10) prior to analyses. Association between rs2736100 and *TP53* gene mutation status was performed using Chi-squared test or Fisher's exact test, with a statistical significance level of *p* = 0.05.

## Results

### Genotyping of rs2736100

All 58 cell lines were successfully genotyped for rs2736100 polymorphism (Table S1). For the samples (*n* = 9) shown as outliers in the Taqman assay, PCR-sequencing was performed to confirm the genotypes. Genotypes of all 9 samples were concordant with the Taqman assay (data not shown). The frequency of the AA, AC, and CC genotypes were 0.22, 0.48, and 0.29, respectively. This result was very comparable to the Hapmap data which is 0.22, 0.52, and 0.27 (Chi-square test, *df* = 2, *p* = 0.88). No significant deviation from the Hardy–Weinberg Equilibrium was observed (Chi-square test, *df* = 2, *p* = 0.79).

### Quantification of relative telomere length (RTL)

The RTL was successfully quantified in 50 out 58 cell lines. Measurement in eight cell lines was failed due to the low DNA quality, thus removed from the final analysis. Among the 50 cell lines, the median and min-max RTL is 0.79, 0.12–23.66. Two melanoma cell lines LOX IMVI and SK-MEL-28 were demonstrated to possess extremely long RTL (23.66 and 6.65, respectively) compared to other cells (Table S1).

### Association between rs2736100 and response to telomerase inhibitors

**Figure 1:**
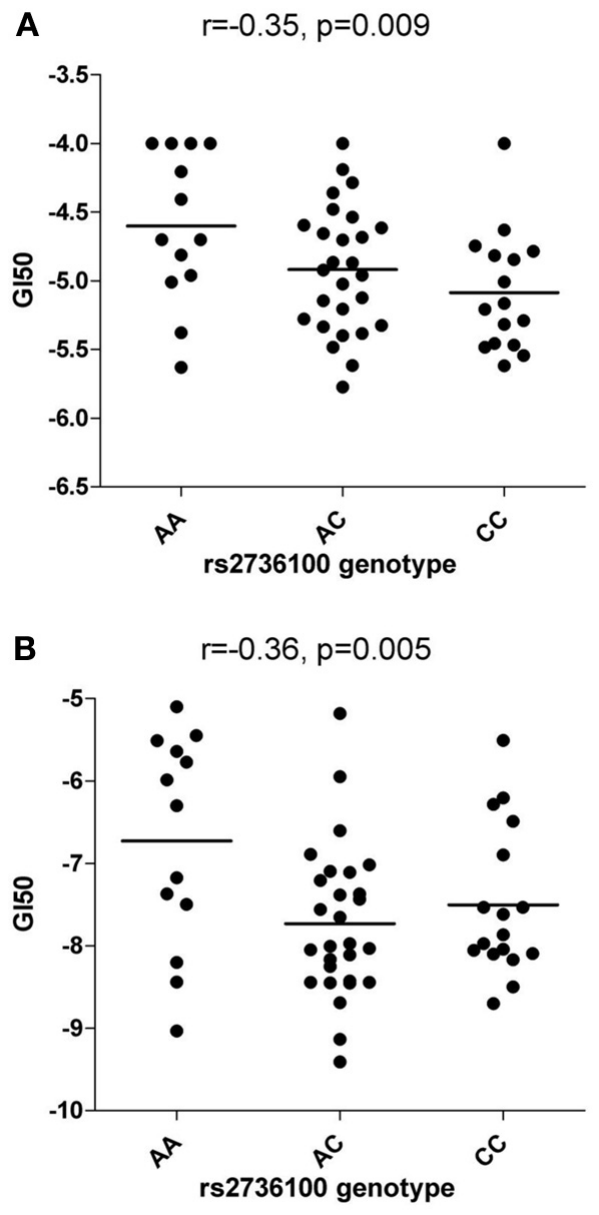
**Correlations between rs2736100 and cytotoxicity of RHPS4/BRACO19 (A) and paclitaxel (B).** SNP genotype was correlated with drug cytotoxicity data by assigning each genotype (AA, AC, and CC) to 0, 1, and 2 (additive model), 0, 0, and 1 (receive model) or 0, 1, and 1 (dominant model) according to the number of C allele. The horizontal bars indicate the mean value of each group. Linear correlation coefficient (r) and *p*-value are indicated as well.

Rs2736100 was significantly associated with sensitivity to RHPS4/BRACO19 (NSC714178) (ANOVA *p* = 0.027) with an additive effect of the C allele (*r* = −0.35, *p* = 0.009) (Figure 1A) but not with Geldanamycin (NSC330507) (ANOVA *p* = 0.69) (data not shown).

### Association between rs2736100 and other anti-cancer agents

**Figure 2:**
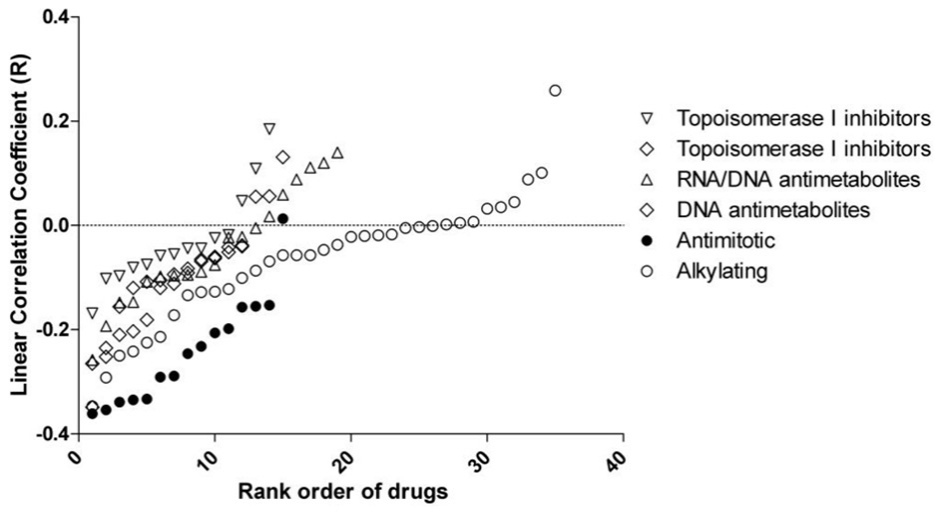
**Linear correlation coefficient (r) between rs2736100 and cytotoxicity of 110 anti-cancer drugs grouped in 6 mechanisms of action.** The figure showed here that compared to other groups of drugs, the GI50s of anti-mitotics were consistently and negatively correlated with rs2736100 genotype in an additive model (0, 1, and 2 corresponding to AA, AC, and CC), suggesting an association between the C allele and increased sensitivity to anti-mitotics.

Significant associations between rs2736100 and sensitivity to 4 out of 15 antimitotic agents were observed (ANOVA, NSC83265 *p* = 0.032; NSC125973 *p* = 0.017; NSC671867 *p* = 0.026; and NSC673188, *p* = 0.039). This was in contrast to only 2 significant associations among the remaining 95 drugs (NSC79037 *p* = 0.014 and NSC755 *p* = 0.026), suggesting an overall association between the polymorphism and anti-mitotic agents (Fisher's exact test, *p* = 0.003). Consistently, there were also marginal associations between the SNP and 3 additional anti-mitotic agents (NSC153858 *p* = 0.058; NSC656178 *p* = 0.074; and NSC666608 *p* = 0.069). *Post-hoc* linear regression analysis demonstrated a significant dominant effect of the C allele on the response to the aforementioned 7 antimitotic drugs, again contrasting to only 3 significant/marginal associations among the remaining 95 drugs (Fisher's exact test, *p* < 0.0001). The linear correlation coefficient values were plotted in Figure 2, where a consistent negative correlation between C allele and sensitivity to all anti-mitotic drugs compared to other groups of agents were demonstrated. Among all anti-mitotic drugs, the most significant association was observed between rs2736100 and paclitaxel (NSC125973, *r* = −0.36, *p* = 0.005) (Figure 1B).

### Correlation between RTL and rs2736100 and cytotoxicity data

No significant association between rs2736100 and RTL was found. There was also no significant correlation between RTL and cytotoxicity data for any group of anti-cancer agents (*p* > 0.05, data not shown).

### Association between rs2736100 and *TP53* mutations

According to the previous study (Ikediobi et al., 2006), *TP53* mutations were found in 42 cell lines, including 12 heterozygous and 30 homozygous mutations. There was a significant association between rs2736100 and the *TP53* mutation status in the NCI-60 panel (overall Chi-squared test in the 3 × 3 table *p* = 0.03), with 82% (37 out of 45) of C-allele carriers (AC + CC) harboring mutations (heterozygous + homozygous) as compared to only 38% (5 out of 13) of the AA genotype (Fisher's exact test, *OR* = 7.4, 95% *CI* = 1.9–28.7, *p* = 0.004) (Table 1).

**Table 1:** **Association between rs2736100 and *TP53* mutations**.

***TP53* mut**	**rs2736100 genotypes**		
	**AA**	**AC**	**CC**
wt	8 (62%)	4 (14%)	4 (24%)
het	2 (15%)	7 (25%)	3 (18%)
hom	3 (23%)	17 (61%)	10 (59%)

*Showing here are the number and percentage of samples with different mutation status among each of the three genotypes. Mut, mutation; wt, wildtype; het, heterozygous; hom, homozygous*.

## Discussion

The genetic basis underlying susceptibility to sensitivity and resistance to anti-cancer treatment remains largely unknown. Identifying genetic factors conferring this susceptibility may lead to personalized treatment for cancers. Our results suggested that a common risk allele increasing susceptibility to various cancers might also influence the efficacy of anti-telomerase and anti-mitotic agents.

The C allele of rs2736100 has been associated with increased risk for multiple cancers, including lung, bladder, pancreatic cancer and glioma (Shete et al., 2009; Hu et al., 2011; Kinnersley et al., 2012; Mocellin et al., 2012; Zou et al., 2012). How this allele functions and increases cancer risk remains incompletely understood. Although it is possible that other SNPs in linkage disequilibrium (LD) with this SNP may be more causal to the overall effect, a bioinformatics study suggested that rs2736100 may be located in a regulatory region of the *hTERT* gene (Zou et al., 2012). Most significantly, the C allele was highly associated with longer telomere length of germ-cells in a few recent studies including a genome-wide association study (Melin et al., 2012; Codd et al., 2013). Since the *hTERT* gene is widely activated in most cancers compared to normal cells, it is highly likely that during carcinogenesis the C allele, and/or its LD proxies, increases the hTERT expression and subsequently confer a higher telomerase activity. Our results demonstrated that the C allele increases the risk for the development of p53 mutations in cancer cells, suggesting that this allele and the associated increased hTERT activity may be beneficial to mutagenesis, thus potentially explaining the association between this allele and the susceptibility to multiple cancers. Further investigation will require a detailed identification of all polymorphisms around the rs2736100 region followed by a mechanistic evaluation of functional pathways that these polymorphisms may alter. However, no significant association between rs2736100 and RTL was found in our dataset. While this might be due to the limited power attributed to the small sample size (*n* = 50), it is also possible that the telomere length of transformed cells might be significantly different from the original germ-cell.

Our study observed a significant correlation between the rs2736100 C allele and sensitivity to a G-quadruplex stabilizer RHPS4/BRACO19, but not Geldanamycin, a HSP90 inhibitor. This discrepancy may reflect the difference in the mechanism of action of the two drugs. While G-quadruplex stabilizers block access of telomerase to telomeres, HSP90 inhibitors prevent the assembly of telomerase-associated proteins required for *hTERT* promoter activity (Ruden and Puri, 2013). However, HSP90 is implicated in many other processes that contribute to carcinogenesis.

Our results also demonstrated that the rs2736100 C allele may specifically influence the cellular response to anti-mitotic agents compared to other commonly used drugs. Anti-mitotics are drugs that disrupt mitotic progression, and have been used extensively for the treatment of cancer (Gascoigne and Taylor, 2009). Anti-mitotics inhibit the function of microtubules by either binding to their subunits or by preventing their growth (Lee et al., 2004). In both cases normal mitotic spindle formation is disrupted, causing cell cycle arrest and eventually apoptosis (Blagosklonny and Fojo, 1999; Park et al., 2011). Previous studies have indicated that the *hTERT* gene might be involved in the spindle assembly checkpoint. This effect may or may not necessarily be confined to hTERT's function in maintaining telomere length (Belivaeu et al., 2007). This is consistent with our observation that there was no significant correlation between RTL of these cells and their response to anti-cancer agents. The telomere length-independent activity of hTERT has been widely observed in cancer cells, e.g., immunofluroescent staining for hTERT in human breast cancer cells has showed that hTERT might be potentially involved in the G2/M mitotic spindle function and checkpoint in mitosis (Belivaeu et al., 2007), whereas activation of a functional spindle assembly checkpoint is required for paclitaxel-induced cell death (Sudo et al., 2004; Yamada and Gorbsky, 2006). Furthermore, as the most commonly mutated gene in various human cancers, *TP53* plays critical roles in mitosis by acting as the cell cycle G1 phase checkpoint and by regulating the spindle checkpoint (Fang and Zhang, 2011). The strong association between rs2736100 and *TP53* mutations in our study suggests that rs2736100 may be prone to retain *TP53* mutagenesis during cancer development, while mutated p53 might mediate at least in part the association between rs2736100 and increased sensitivity to anti-mitotics. Our finding is consistent with previous observations, e.g., ectopic expression of hTERT in HMECs resulted in reduced basal level of active p53 (Belivaeu et al., 2007), while p53 deficient RKO colorectal cancer cells were four times more sensitive to paclitaxel compared to wild type RKO cells (Rakovitch et al., 1999). Nevertheless, as thus far the hTERT function in carcinogenesis, its interaction with other molecules as well as its activity independent of the telomere maintenance are still poorly understood, further investigation will be necessary to completely elucidate the mechanism underlying the observations in our study.

In conclusion, our study suggests that inherently increased hTERT function may play an important role in cancer development and may also affect efficacy of anti-cancer agents. Continued studies in both the *in vitro* and *in vivo* settings are warranted.

### Conflict of interest statement

The authors declare that the research was conducted in the absence of any commercial or financial relationships that could be construed as a potential conflict of interest.
