# Supplementary material for: Association between hTERT rs2736100 polymorphism and sensitivity to anti-cancer agents
Source: Front Genet. 2013 Aug 26;4:162. doi: 10.3389/fgene.2013.00162 (PMC3752523; doi:10.3389/fgene.2013.00162)
Supplement: Supplementary file 1 [file 49411_Liu_DataSheet1.DOCX]

**SUPPLEMENT MATERIALS**

**Association Between hTERT rs2736100 Polymorphism and Sensitivity to Anti-cancer Agents**

Julie Kim^1^, Yava L. Jones-Hall^2^, Rongrong Wei^1^, Jamie Myers^3^, Yuan Qi^4^, Gregory T. Knipp^5^ and Wanqing Liu^1,6*^

^1^Department of Medicinal Chemistry and Molecular Pharmacology, College of Pharmacy, Purdue University, West Lafayette, IN 47907

^2^Department of Comparative Pathobiology, College of Veterinary Medicine, Purdue University, West Lafayette, IN 47907

^3^Department of Medicine, The University of Chicago, Chicago, IL 60637

^4^Department of Computer Science, Purdue University, West Lafayette, IN 47907

^5^Department of Industrial and Physical Pharmacy, College of Pharmacy, Purdue University, West Lafayette, IN 47907

^6^Purdue University Center for Cancer Research, West Lafayette, IN 47907

Running Title: TERT polymorphism and sensitivity to anticancer drugs

Submitted to: Frontiers in Pharmacogenetics and Pharmacogenomics

*Correspondence: Wanqing Liu, Ph.D, Department of Medicinal Chemistry and Molecular Pharmacology, College of Pharmacy, Purdue University; 575 Stadium Mall Dr, West Lafayette, IN 47907, USA. Tel: 1-765-496-6389; Fax: 1-765-494-1414; Email: [liu781@purdue.edu](mailto:liu781@purdue.edu).

Table S1. Genotype of rs2736100 and relative telomere length (RTL) of the NCI-60 cancer cells.

| **Panel** | **Cell Name** | **rs2736100 Genotype** | **RTL** |
| --- | --- | --- | --- |
| Breast | BT-549 | AC | 0.29 |
| Breast | HS 578T | CC | 1.23 |
| Breast | MCF7 | CC | 0.72 |
| Breast | MDA-MB-231 | AC | 0.38 |
| Breast | MDA-MB-435 | AC |  |
| Breast | NCI/ADR-RES | CC | 1.26 |
| Breast | T-47D | CC | 0.62 |
| Central Nervous System | SF-268 | AC | 1.69 |
| Central Nervous System | SF-295 | AC | 0.40 |
| Central Nervous System | SF-539 | CC |  |
| Central Nervous System | SNB-19 | AC | 1.30 |
| Central Nervous System | SNB-75 | AC | 0.58 |
| Central Nervous System | U-251 | AC | 1.96 |
| Colon | COLO 205 | CC | 0.45 |
| Colon | HCC-2998 | AC | 0.55 |
| Colon | HCT-116 | AA | 1.11 |
| Colon | HCT-15 | AC | 1.30 |
| Colon | HT-29 | AC | 1.49 |
| Colon | KM-12 | CC | 0.44 |
| Colon | SW-620 | AC | 0.28 |
| Leukemia | CCRF-CEM | AC | 1.82 |
| Leukemia | HL-60 | AC | 1.11 |
| Leukemia | K-562 | AC | 0.74 |
| Leukemia | MOLT-4 | AC | 1.56 |
| Leukemia | RPMI-8226 | CC | 0.21 |
| Leukemia | SR | AC |  |
| Melanoma | LOX IMVI | AC | 23.66 |
| Melanoma | M14 | CC | 1.64 |
| Melanoma | MALME-3M | AA | 0.84 |
| Melanoma | SK-MEL-2 | AA | 0.87 |
| Melanoma | SK-MEL-28 | AA | 6.65 |
| Melanoma | SK-MEL-5 | AC |  |
| Melanoma | UACC-257 | CC | 0.35 |
| Melanoma | UACC-62 | CC |  |
| Non-Small Cell Lung | A549 | AC | 1.09 |
| Non-Small Cell Lung | EKVX | AA | 0.56 |
| Non-Small Cell Lung | HOP-62 | CC | 0.68 |
| Non-Small Cell Lung | HOP-92 | CC | 1.26 |
| Non-Small Cell Lung | NCI-H226 | CC | 0.51 |
| Non-Small Cell Lung | NCI-H23 | AC | 4.87 |
| Non-Small Cell Lung | NCI-H322M | AC |  |
| Non-Small Cell Lung | NCI-H460 | AA | 1.20 |
| Non-Small Cell Lung | NCI-H522 | CC | 0.12 |
| Ovarian | IGR-OV1 | AA | 0.95 |
| Ovarian | OVCAR-3 | AC |  |
| Ovarian | OVCAR-4 | AC | 1.66 |
| Ovarian | OVCAR-5 | AA | 0.60 |
| Ovarian | OVCAR-8 | CC | 1.37 |
| Ovarian | SK-OV-3 | AC | 3.30 |
| Prostate | DU-145 | CC | 0.50 |
| Prostate | PC-3 | AC | 0.48 |
| Renal | 786-0 | AC | 0.26 |
| Renal | A498 | AA | 0.27 |
| Renal | ACHN | AA | 0.21 |
| Renal | CAKI-1 | AA | 0.37 |
| Renal | SN12C | AA | 2.92 |
| Renal | TK-10 | AC |  |
| Renal | UO-31 | AA | 0.30 |
